# Characterization of the complete chloroplast genome sequence of *Anisodus acutangulus* (Solanaceae)

**DOI:** 10.1080/23802359.2020.1717387

**Published:** 2020-01-27

**Authors:** Xing Tian, Ji-Qing Bai, Cong-Wei Yang, Ying-Min Zhang, Guo-Dong Li

**Affiliations:** a Faculty of Traditional Chinese Pharmacy, Yunnan University of Chinese Medicine, Kunming, China;; b College of Pharmacy, Shanxi University of Chinese Medicine, Xianyang, China

**Keywords:** *Anisodus acutangulus*, Solanaceae, chloroplast genome, phylogenetic analysis

## Abstract

*Anisodus acutangulus* is a Solanaceae perennial plant, which is endemic to China and classified as an endangered species. In this study, we have sequenced the complete chloroplast genome of *A. acutangulus*, which is 156,079 bp in length, containing a large single-copy (LSC) region of 86,526 bp, a small single-copy (SSC) region of 17,741 bp and comprises a pair of inverted repeat regions (IRs) of 25,906 bp. Totally 134 genes were annotated, including 87 protein-coding genes, 39 tRNA genes, and 8 rRNA genes. Its overall GC content is 37.6%. Phylogenetic analysis using total chloroplast genome DNA sequence of 21 species revealed that *A. acutangulus* was closely related to *Hyoscyamus niger* with 100% bootstrap value.

*Anisodus acutangulus* Wu and Chen is a perennial herb belonging to the Solanaceae family, endemic to China, and it is distributed in the elevation of 2750–3000 m of the Grassy slopes and waste lands (Flora of China Editorial Committee of Chinese Academy of Sciences 1994). It has been used as an anesthetic in therapeutics for centuries (Kai et al. 2012). In addition, it has an excellent pharmacological activity and was used to treat fractures, arthralgia, stomachache, intestinal colic, and so on (Yang and Li 2013). Owing to the over-exploitation and several anthropogenic factors, the wild population of *A. acutangulus* decreased rapidly in recent years. *Anisodus acutangulus* has been categorized as a Critically Endangered (CR) species in the Red List of Chinese Plants (Qin et al. 2017). In this study, we reported and characterized the complete chloroplast genome sequence of *A. acutangulus* based on the next-generation sequencing. The plastome of *A. acutangulus* reported here will provide informative data both for the conservation genetics of this species and for the evolutionary study of genus *Anisodus*.

The fresh leaves of *A. acutangulus* were collected from Dali city (25°52′N, 100°00′E), Yunnan province, China, and the voucher specimens (5329010384) are stored in the Herbarium of Yunnan University of Chinese Medicine. Total genomic DNA was extracted using plant DNA (Bioteke Corporation, China), then we sequenced the complete chloroplast genome using the lllumina Hiseq 2500 platform (lllumina Inc., SanDiego, CA). Totally 3.6 GB of reads were generated and the sequence was assembled from the scratch using NOVOPlasty (Dierckxsens et al. 2017). Finally, the complete chloroplast genome was annotated with the online annotation tool GeSeq (Tillich et al. 2017), and Geneious R11 11.1.5 (Biomatters Ltd., Auckland, New Zealand) to correct the annotation.

The chloroplast genome size of *A. acutangulus* is 156,079 bp (GenBank accession No.: MN781973), containing a large single-copy (LSC) region of 86,526 bp, the small single-copy (SSC) region of 17,741 bp, and comprises a pair of inverted repeat regions (IR) of 25,906 bp. Totally 134 genes were annotated, including 87 protein-coding genes, 39 tRNA genes, and 8 rRNA genes. The overall GC content of *A. acutangulus* chloroplast genome is 37.6%, and LSC, SSC, and IR regions are 35.6%, 31.8%, and 42.9%, respectively. The number of mono-, di-, tri-, tetra-, penta-, and hexa- nucleotides SSRs are 43, 8, 2, 7, 1, and 0, respectively.

To perform phylogenetic analysis and determine the phylogenetic location of *A. acutangulus*, we selected the complete chloroplast genomes of 21 species, including 19 Solanaceae species and one species each of Acanthaceae and Convolvulaceae as outgroups. All chloroplast genomes were compared by MAFFT v.7 (Katoh and Standley 2013) and phylogenetic trees were generated using GTR models performed with RaxML (Stamatakis 2014) using 1000 bootstrap replicates. Phylogenetic analysis using total chloroplast genome DNA sequence of 21 species revealed that *A. acutangulus* was closely related to *Hyoscyamus niger* (Figure 1). The characterization of *A. acutangulus* complete chloroplast genome provides useful data for resource conservation and future genetic studies of the species.

**Figure 1.**
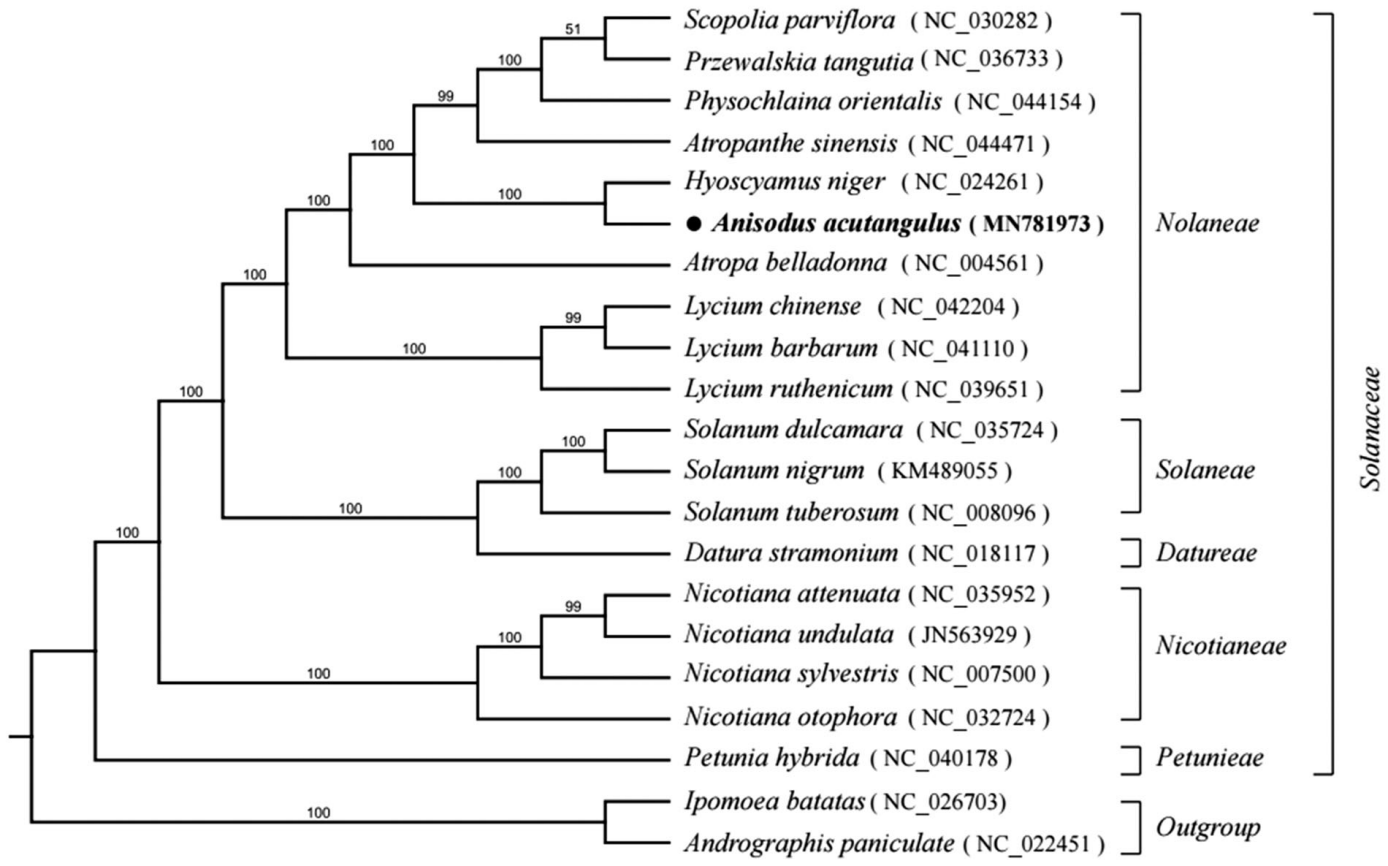
Maximum likelihood phylogenetic tree inferred from 21 chloroplast genomes. Bootstrap support values >50% are indicated next to the branches (GenBank accession No.: MN781973).
